# Clinical and economic burden of organ damage among patients with systemic lupus erythematosus in a real-world setting in Germany

**DOI:** 10.1186/s41927-024-00387-6

**Published:** 2024-05-17

**Authors:** Michael Schultze, Elena Garal-Pantaler, Marc Pignot, Roger A Levy, Heike Carnarius, Matthias Schneider, Kerry Gairy

**Affiliations:** 1 Berlin Center for Epidemiology and Health Research, ZEG Berlin GmbH, Invalidenstr. 115, 10115 Berlin, Germany; 2 Health Care Research and Health Economics (Versorgungsforschung und Gesundheitsökonomie), Team Gesundheit GmbH, Rellinghauser Straße 93, 45128 Essen, Germany; 3 Global Medical Affairs, GSK, 1250 S Collegeville Rd, Collegeville, PA 19426 USA; 4 Specialty Care Medical Affairs, GSK GmbH & Co. KG, Heidenkampsweg 51, 20097 Hamburg, Germany; 5 Policlinic and Hiller Research Unit for Rheumatology, Medical Faculty, University Hospital Düsseldorf, Heinrich Heine University Düsseldorf, Moorenstr 5, 40225 Düsseldorf, Germany; 6 GSK, Value Evidence and Outcomes, 980 Great West Road, Brentford, Middlesex TW8 9GS UK

**Keywords:** Organ damage, Systemic lupus erythematosus, Real-world evidence, Healthcare resource utilization, SLICC/ACR damage index, Economic burden

## Abstract

**Background:**

Systemic lupus erythematosus (SLE), a chronic multisystem autoimmune disease, carries high risk of organ damage and burden to healthcare systems. SLE disease modification aims to reduce disease activity with minimal treatment toxicity and preventing or minimizing organ damage development. This real-world study utilizing healthcare administrative claims data assessed organ damage development, associated costs and healthcare resource utilization (HCRU) in patients with SLE in Germany.

**Methods:**

Claims data from January 1, 2007, to December 31, 2017, were obtained from the Betriebskrankenkassen German Sickness Fund Database. Adults (> 18 years) with a confirmed SLE diagnosis between January 1, 2009, and December 31, 2014, (inclusion period) were included. The index date was calculated based on the first recorded SLE diagnosis during this period. Patients were propensity score–matched (1:3) to a comparator cohort without SLE by age, sex, and comorbidities (Charlson comorbidity index). Organ damage was identified using an algorithm developed based on conditions described in the Systemic Lupus International Collaborating Clinics/American College of Rheumatology Damage Index (SDI), using ICD-10-GM diagnostic codes, healthcare procedures, and/or treatments.

**Results:**

2121 patients with SLE and 6308 comparator patients were included (mean follow-up time: 6.4 years). Organ damage prevalence increased from 60.5% at baseline to 83.0% during 6 years of follow-up in all patients with SLE, while 17.0% of patients with SLE did not develop organ damage. Patients with newly confirmed SLE diagnosis without organ damage at baseline were nearly twice as likely to develop organ damage within 5 years versus the comparator cohort (52.0% vs. 27.0%). Total annual costs per patient-year for patients with SLE with organ damage were more than double those of patients with SLE without organ damage; both the number of inpatient admissions and length of stay were higher.

**Conclusions:**

The application of a recently developed algorithm allowed us to use claims data to elucidate SLE organ damage, and its associated high clinical and economic burden, in a large, representative sample in Germany. To our knowledge, this is the first European analysis of its kind involving a broad cohort of patients with SLE treated in the routine care setting.

**Supplementary Information:**

The online version contains supplementary material available at 10.1186/s41927-024-00387-6.

## Background

Although survival rates among patients with systemic lupus erythematosus (SLE) have increased in recent decades, more than half of patients develop irreversible organ damage within 10 years of SLE diagnosis, and this continues to increase by 15 years [1–6] resulting in significant additional morbidity and early mortality [5]. Commonly damaged organ domains include ocular, musculoskeletal, renal, cutaneous, cardiovascular, and the central nervous system [2, 4].

European Alliance of Associations for Rheumatology (EULAR) guidelines for the management of SLE recommend that organ damage should be measured annually using the Systemic Lupus International Collaborating Clinics (SLICC)/American College of Rheumatology (ACR) Damage Index (SDI) [7, 8]. The SDI includes 42 items for 12 organ systems, with a maximum score of 46, and any increment in SDI is clinically and prognostically significant [7]. However, this assessment requires long-term follow-up and is rarely captured in secondary data, such as administrative claims data, which makes the clinical and economic burden of organ damage difficult to quantify from these data sources. Indeed, while determinants of organ damage in SLE have been examined in controlled clinical trials [9] and organ damage progression has been assessed in single-arm, open-label extension studies [10], real-world studies of organ damage in routine clinical practice are more limited, particularly outside academic, specialist centers [11, 12].

Administrative claims data are one source of real-world data that are intended for non-research purposes, usually for billing; therefore, claims databases only contain information pertinent to that purpose, as opposed to data from clinical trials or registries which are intended for research purposes [13]. Further, analyses of claims data are not based on data from specific physicians/centers, and patients are included based on diagnosis, with the frequency of data collection based on their routine attendance to treating physicians. As a result, administrative claims data do not contain records of, for example, SLE disease activity measures or SDI, data pertaining to ACR/EULAR criteria for SLE classification [14], or indirect healthcare data. However, an advantage of using administrative claims data is the access to large data samples from a broadly representative population, with diagnoses and organ damage accurately captured, allowing investigations into whether observations in specific SLE registries remain true in more generalizable cohorts. Therefore, our aim was to identify manifestations of organ damage, as described in the SDI, in administrative claims data.

A number of country-specific studies have evaluated the costs associated with organ damage in SLE [15–21]; however, of these, only Jönsen et al. 2015 included a control population of patients without SLE for comparison. The lack of real-world data on the burden of organ damage in SLE may be partially due to the limited use of the SDI in clinical settings [22–24]. The SDI is not validated beyond SLE, hindering any direct comparison to other populations, but by recording the presence of conditions listed in the SDI a similar view of morbidity can be achieved in SLE and non-SLE cohorts. Thus, a deeper understanding of the real-world burden of organ damage is important to quantify treatment benefits, particularly with the increasing use of disease-modifying therapies.

This non-interventional analysis (GSK study 209523) used healthcare administrative claims data to assess the evolution of organ damage and associated costs and healthcare resource utilization (HCRU) among patients with SLE in Germany; this included describing the healthcare system’s economic burden of organ damage in patients with SLE compared with matched comparator patients without SLE, as well as with patients with SLE and no organ damage. We also investigate burden of organ damage in patients with a newly confirmed SLE diagnosis compared with those with a pre-existing diagnosis of SLE.

## Methods

### Data source

Claims data from January 1, 2007, to December 31, 2017, were obtained from the Betriebskrankenkassen (BKK) German Sickness Fund Database. BKK is a representative database from approximately 72 million patients covered by statutory health insurance (Gesetzliche Krankenversicherung; GKV) in Germany, which in 2017 included 5,067,249 individuals available for this study.

### Study design

**Fig. 1:**
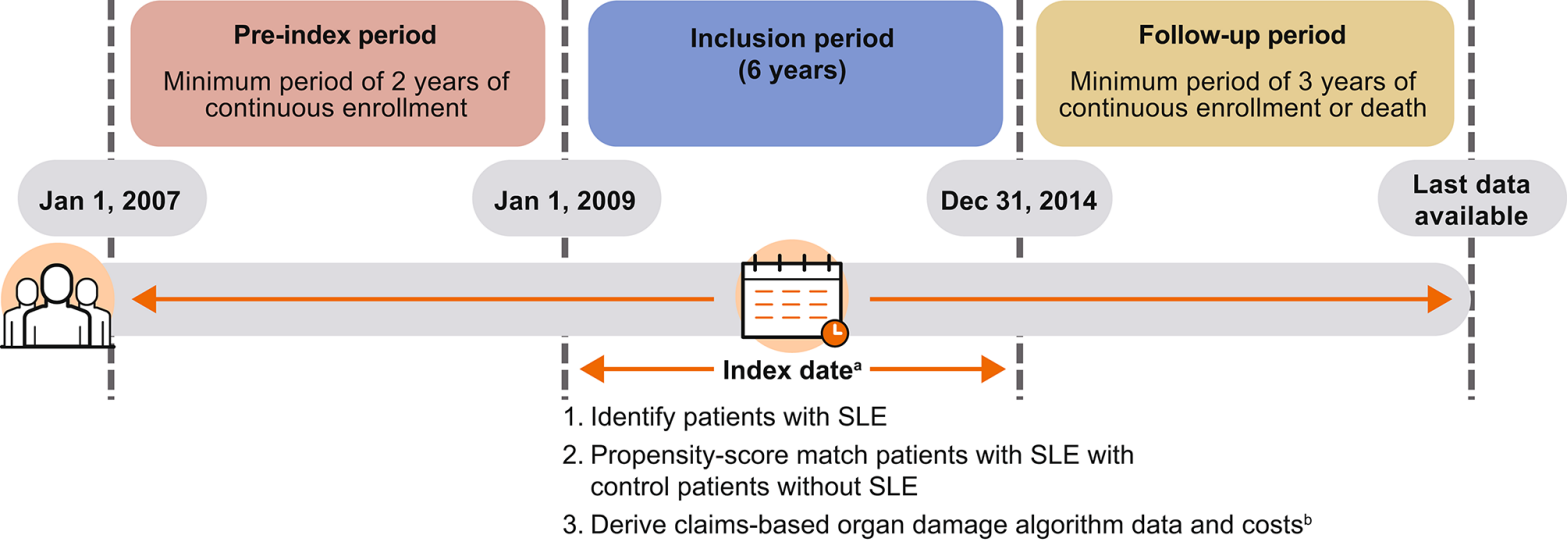
Study design ^a^First day of the first quarter with the first recorded SLE diagnosis identified during the inclusion period (i.e. by hospital discharge or by outpatient claim); ^b^Including data from pre-index period SLE: systemic lupus erythematosus

Adult patients classified with SLE between January 1, 2009, and December 31, 2014, (the inclusion period) were included (Fig. 1). The index date was defined as the first day of the first quarter with the first recorded SLE diagnosis identified during the inclusion period (by hospital admission or by outpatient claim).

Adult patients with a confirmed SLE diagnosis during the inclusion period were propensity score–matched (1:3) to a comparator cohort without SLE by age, sex, categories of comorbid conditions per Charlson comorbidity index (CCI), and CCI score [25]. Comorbidities were matched to identify a population with similar clinical burden, thus allowing the incremental impact of conditions listed in the SDI to be ascertained, since many of these conditions are not specific to SLE. Propensity score was calculated by multivariate logistic regression.

### Study population

Eligible patients comprised those > 18 years of age at index date and with ≥ 1 confirmed diagnosis of SLE via inpatient- or outpatient-based claims during the inclusion period, using International Classification of Diseases, 10th Revision, German Modification (ICD-10-GM) codes M32.1 (SLE with organ or system involvement), M32.8 (other forms of SLE), or M32.9 (SLE, unspecified); EULAR/ACR classification criteria for SLE were not used to confirm diagnoses as these criteria are not captured in claims data. We developed an algorithm to verify the SLE diagnoses using available data in the German health insurance BKK database: inpatient-based confirmation of an SLE diagnosis was defined as ≥ 1 hospital discharge claim or an ambulatory visit claim. Outpatient-based confirmation was defined in patients without hospital discharge claims as ≥ 1 outpatient claim reported by a specialist and ≥ 1 prescription of one of the following drugs during follow-up after the first documented SLE diagnosis during the study period: hydroxychloroquine, chloroquine, azathioprine, methotrexate, mycophenolate mofetil or mycophenolic acid, belimumab, rituximab, cyclophosphamide, cyclosporine A, tacrolimus, or glucocorticoids for systemic use. Eligible patients were also required to have had continuous enrollment in the database for ≥ 2 years before the index date (pre-index period), and continuous enrollment for ≥ 3 years after the index date (during the follow-up period), except in the case of death.

Patients were categorized into subgroups according to presence or absence of organ damage. In addition, within the SLE cohort, subgroups were also defined based on presence or absence of an SLE diagnosis during the pre-index period (termed pre-index SLE and newly confirmed SLE diagnosis, respectively).

### Comparator population

The comparator cohort was a randomly selected group of patients without an SLE diagnosis who were identified and matched to the patients with SLE meeting the study eligibility criteria, based on demographics and comorbidities per CCI. Propensity score matching was performed to eliminate the impact of confounding parameters, thus avoiding any potential imbalance in patient demographics or comorbidities between patients with and without SLE. To ensure the comparator cohort had no history of SLE, these patients were required to have continuous database enrollment and an SLE diagnosis-free record during the entire study period. Comparator cohort patients were assigned the index date and the end of the follow-up date of their matched case patient with SLE.

### Definition of organ damage

To identify organ damage resulting from SLE in a statutory health insurance database, an algorithm was developed based on the conditions listed in the SDI, using ICD-10-GM diagnostic codes, healthcare procedures, or treatments, as claims databases do not contain identifiable data as defined by SDI. Medical conditions scored in the SDI must have been present for at least 6 months to be characterized as irreversible (unless they are specified as having existed “ever”), which was reflected in our algorithm. Per the actual SDI, in clinical practice, organ damage is scored only if it occurs after the SLE diagnosis; however, SLE and potentially SLE-related organ damage may exist for several years prior to the formal diagnosis. Thus, organ damage was also reported, separately, for newly diagnosed patients with SLE in the pre-index period.

The following criteria qualified a diagnosis as organ damage:

- ICD-10-GM codes:
  - Presence of at least one relevant ICD-10-GM code for the following SDI items: cataract, cerebrovascular accident, coronary artery bypass, myocardial infarction, avascular necrosis, significant tissue loss, infarction or resection of bowel below duodenum, spleen, liver or gall bladder, stricture or upper gastrointestinal tract surgery.
  - For SDI items requiring at least 6 months of persistence, items had at least 2 quarters with ICD codes of the same medical condition, 2 or more (up to 6) quarters apart.
  - For recurrent events (for example, stroke or myocardial infarction), a minimum of 6 months was required between two consecutive claims with the same medical condition to be scored as two individual events.
- Operation and Procedure System (OPS) codes:
  - Presence of at least one OPS code defines damage.
- Anatomical Therapeutic Chemical (ATC) codes:
  - Seizures: ATC code plus respective ICD-10-GM code needed to define damage, requiring at least 6 months of therapy.
  - Pancreatitis: ATC code required to define an insufficiency requiring enzyme replacement, requiring at least 6 months of therapy.

### Study variables

Demographic, clinical, and treatment covariates were retrieved at index date. Baseline CCI was calculated based on conditions observed during the 2-year pre-index period, where each condition was assigned a weight of 1, 2, 3, or 6 based on the condition-associated risk of death; the CCI score was calculated as the sum of the assigned weights [25].

Cumulative organ damage was identified using an algorithm developed based on conditions described in the SDI using ICD-10-GM diagnostic codes, healthcare procedures, and/or treatments (Additional file 1). This organ damage claims algorithm was used in the comparator cohort to allow comparison/estimation of incremental organ damage burden among patients with SLE (i.e. by providing a ‘baseline’ of the relevant conditions that occur regardless of SLE).

The primary outcome measure was the economic burden in terms of direct healthcare costs in the main SLE cohort versus comparator cohort, and patients in the SLE cohort with organ damage versus without organ damage. Direct healthcare costs comprised the total annual costs per patient-year (PY), which consisted of inpatient admissions and ambulatory visits (walk-in hospital services without in-bed stay), outpatient visits, prescription costs, sickness, and other health insurance-funded benefits. Costs were reported in Euros, deflated to 2017 based on the German Consumer Index. Secondary outcomes included HCRU and related costs associated with organ damage, the prevalence and incidence rates of organ damage overall and by SDI organ domain in newly confirmed SLE diagnosis and pre-index SLE subgroups, the time to first onset of organ damage in patients with a newly confirmed SLE diagnosis, and the time to worsening of organ damage in the SLE cohort. HCRU included inpatient admissions, ambulatory visits (i.e. walk-in hospital services without an in-bed stay), outpatient visits, SLE medication, and medical procedures.

### Statistical analyses

Study measures were summarized descriptively. The main comparisons were the matched SLE cohort versus matched non-SLE comparator cohort, and patients with organ damage (defined as all patients with SLE with organ damage pre- or post-index) versus patients without organ damage (defined as all patients with SLE without organ damage pre- or post-index) (Fig. S1 in Additional file 1).

Total annual per PY healthcare costs were compared between the matched SLE and comparator groups using the paired Wilcoxon test. Adjusted Cox regression was used to compare the risk of organ damage events between all SLE and comparator cohort patients. Mean/frequency of HCRU and costs were provided by annual interval for the entire follow-up period and were summarized per patient-year. The association between the occurrence of organ damage and cumulative parameters of medication in the preceding year was tested by logistic regression. Time to organ damage events was displayed graphically using Kaplan–Meier curves and differences between groups were assessed with the log-rank test. Hazard ratios (HR) were calculated by Cox regression for time to first organ damage and worsening of organ damage with covariates of sex, age group, baseline CCI comorbidities, and the additional covariate of baseline medication for time to worsening of organ damage.

## Results

### Patient population

A total of 2121 patients with SLE and 6308 propensity score–matched comparator patients without SLE were included in the study (Fig. S1 in Additional file 1). Between 2009 and 2017, 191 (9.0%) patients with SLE died. Demographics and baseline clinical characteristics of matched cohorts are shown in Table 1. Mean (standard deviation; SD) CCI at baseline was 1.98 (1.92) points in patients with SLE and 1.99 (1.14) in the comparator cohort. Approximately half of the patients (53.0%) in the SLE cohort had a rheumatic disease recorded (as per the CCI definition) in the pre-index period, including patients with a newly confirmed SLE diagnosis with no concomitant rheumatic condition. In the comparator cohort, patients were matched by corresponding pre-index comorbidities, where 52.7% of them had a rheumatic disease other than SLE. The second most common comorbidity was chronic pulmonary disease (30.5% and 30.8% in the SLE and comparator cohorts, respectively; Table 1). Corticosteroids were the most frequently prescribed SLE medication at baseline in the SLE cohort (68.5%), followed by antimalarials (48.2%) and immunosuppressants (27.7%). Within the SLE cohort, 1037 (48.9%) patients were classified as having pre-index SLE and 1084 (51.1%) were classified as having a newly confirmed SLE diagnosis.

**Table 1:** Demographics^a^ and baseline clinical characteristics

	SLE cohort (*N* = 2121)	Comparator cohort (*N* = 6308)
Female, n (%)	1770 (83.45)	5259 (83.37)
Age, mean (SD)	50.86 (16.18)	51.42 (9.39)
Follow-up length, mean (SD), years	6.37 (2.05)	6.36 (2.05)
Pre-index SLE	1037 (48.89)	-
Newly confirmed SLE diagnosis	1084 (51.11)	-
CCI score, mean (SD)	1.98 (1.92)	1.99 (1.14)
**CCI comorbidities in > 5% of patients in either group, n (%)**		
Rheumatic disease^b, c^	1124 (52.99)	3321 (52.65)
Chronic pulmonary disease	647 (30.50)	1943 (30.80)
Mild liver disease	274 (12.92)	806 (12.78)
Cerebrovascular disease	240 (11.32)	730 (11.57)
Renal disease	229 (10.80)	625 (9.91)
Peripheral vascular disease	215 (10.14)	662 (10.49)
Diabetes without chronic complication	173 (8.16)	511 (8.10)
Any malignancy, except malignant neoplasm of the skin	163 (7.69)	503 (7.97)
Congestive heart failure	150 (7.07)	431 (6.83)
**CCI score, n (%)**		
0	450 (21.22)	1350 (21.40)
1	642 (30.27)	1928 (30.56)
2	412 (19.42)	1230 (19.50)
3+	617 (29.09)	1800 (28.54)
**Baseline SLE medications**^**d**^, n (%)		
Antimalarials	1022 (48.2)	-
Corticosteroids	1453 (68.5)	-
Immunosuppressants	588 (27.7)	-
Methotrexate	296 (14.0)	-
Cyclophosphamide	32 (1.5)	-
Sulfasalazine	21 (0.99)	-
Biologics	19 (0.9)	-
Belimumab	6 (0.28)	-

^a^No ethnicity data were available. ^b^Baseline characteristics of the SLE cohort captured during the pre-index period included patients who do not yet have SLE (newly diagnosed subgroup; not all rheumatic disease was SLE). ^c^ICD-10 codes M05.x, M06.x, M31.5, M32.x– M34.x, M35.1, M35.3, M36.0 per CCI definition of rheumatic diseases. ^d^Baseline SLE medication for the SLE cohort only

CCI: Charlson comorbidity index; ICD-10: International Classification of Diseases-10; SD: standard deviation; SLE: systemic lupus erythematosus

Overall, 1760 (83.0%) patients with SLE were identified as having organ damage (either pre-index or during follow-up), and 361 (17.0%) patients with SLE were without organ damage (no organ damage in the pre-index period, and did not develop organ damage during follow-up). Baseline characteristics are shown in Table S1 in Additional file 1. Compared with patients with organ damage, patients without organ damage were younger (mean [SD] 38.43 [10.83] versus 53.41 [15.92] years), and in line with having less organ damage, had lower baseline CCI (mean [SD] 0.85 [0.94] versus 2.21 [1.99]) and had fewer baseline comorbidities.

Among patients with pre-index organ damage (60.5% [*n* = 1283]), the most frequently affected domains included ocular, neuropsychiatric, and cardiovascular and were generally similar between pre-index and newly confirmed SLE diagnosis subgroups.

### Economic burden of organ damage in patients with SLE

**Fig. 2:**
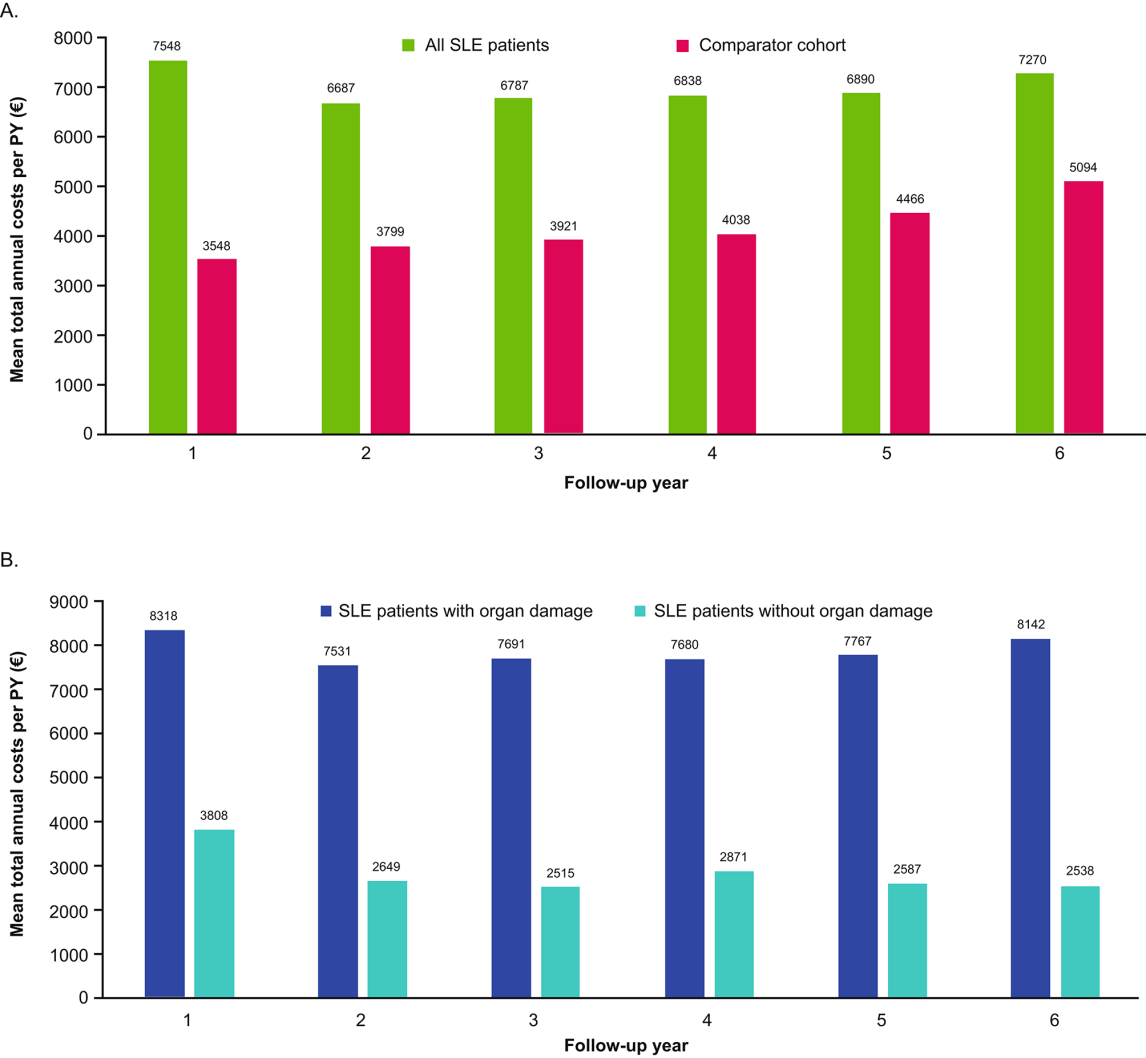
Total healthcare costs (Euro) per patient-year by follow-up year (**A**) All SLE patients and the comparator cohort^a^ and (**B**) patients with SLE with organ damage and without organ damage ^a^Patients with a confirmed SLE diagnosis during the inclusion period were propensity score–matched (1:3) to a comparator cohort without SLE by age, sex, categories of comorbid conditions per CCI, and CCI score CCI: Charlson comorbidity index; PY: patient-year; SLE: systemic lupus erythematosus

Total annual costs per PY for patients with SLE for each year of follow-up were significantly higher than those of the comparator cohort despite morbidity matching (*p* < 0.0001 in Years 1–5 and *p* = 0.0412 in Year 6) (Fig. 2A). PY costs for the entire SLE cohort (those with and without organ damage) exceeded those of the morbidity-matched comparator cohort at all time points (e.g. SLE vs. controls, Year 1: €7548 vs. €3548; Year 6: €7270 vs. €5094). Total annual costs per PY for patients with SLE and organ damage were more than double those of patients with SLE without organ damage in the first follow-up year (€8318 vs. €3808; factor of 2.18), with a similar trend observed in the subsequent follow-up years (Fig. 2B). The difference in expenditure between patients with SLE with and without organ damage increased over time, with total annual costs per PY for patients with organ damage reaching 3.2 times those of patients without organ damage in Year 6. Given the difference in age between cohorts at baseline, a sensitivity analysis matching the subgroups with and without organ damage by age and sex was performed, which demonstrated that the PY costs for patients were 2.07 times those of patients without organ damage. As the sensitivity analyses found costs per PY of the subgroup with organ damage still exceeded those of the subgroup without organ damage, these analyses report on unadjusted data.

During the follow-up period, costs associated with inpatient admissions were the major contributors to the total costs in both SLE with organ damage and SLE without organ damage subgroups, followed by pharmacy costs and outpatient visits (Figure S2. Inpatient costs were considerably higher in patients with organ damage than in patients without organ damage, with the largest cost occurring in Year 1 (€3627.54 and €1376.81 per PY, respectively; Figure S2) and the greatest difference between organ damage subgroups occurring in Year 3 (€3044.75 and €560.90 per PY, respectively; factor of 5.43).

### HCRU

During the follow-up period, among patients of the SLE with organ damage subgroup, both the number of inpatient admissions and length of stay were higher than among patients of the SLE without organ damage subgroup (Table S2). In the overall SLE population, the most common non-SLE reasons for inpatient admissions were pneumonia (5.4% [*n* = 92]), heart failure (5.2% [*n* = 88]), and angina pectoris (4.3% [*n* = 71]; Table S3).

Detailed all-cause healthcare costs and HCRU for the SLE subgroups are presented in Additional file 1.

In the overall SLE population, 62% (*n* = 1314) of patients received antimalarials during follow-up, 38% (*n* = 799) received immunosuppressants, 89% (*n* = 1889) received corticosteroids, 23% (*n* = 488) received methotrexate, and 6% (*n* = 121) of patients received a biologic agent. Of all treatments, corticosteroids were received for the longest duration in all subgroups, especially in the first year of the follow-up period, with longer durations in patients with organ damage than those without (Figure S3). The duration of immunosuppressants intake was longer in patients with organ damage than those without organ damage.

### Clinical burden of organ damage in patients with SLE

**Fig. 3:**
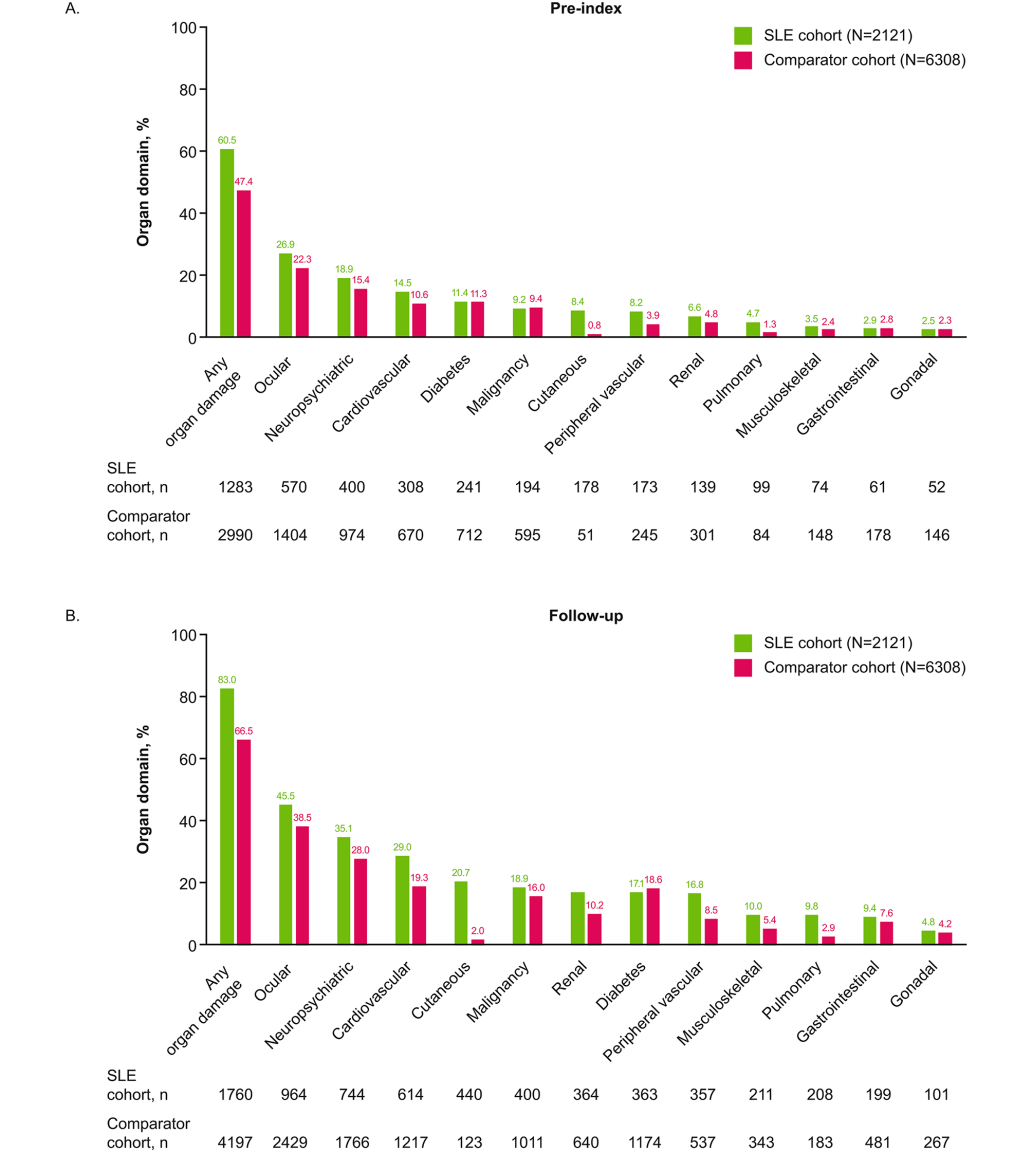
Most commonly affected organ domains pre-index (**A**) and at follow-up (**B**) SLE: systemic lupus erythematosus

Baseline (pre-index) organ damage prevalence (indicated by ≥ 1 pre-index SDI condition) was 60.5% in the SLE cohort (Fig. 3A). The pre-index SLE and newly confirmed SLE diagnosis subgroups showed differing prevalence of organ damage at baseline; pre-index organ damage was present in 66.0% (*n* = 684/1037) of patients with pre-index SLE, compared with 55.3% (*n* = 599/1084) of patients with a newly confirmed SLE diagnosis.

During follow-up, the proportion of patients with organ damage in the SLE cohort increased to 83.0% (compared with 66.5% of patients with organ damage in the comparator cohort; Fig. 3B). Again, the pre-index SLE and newly confirmed SLE diagnosis subgroups differed in organ damage prevalence, with 86.9% (*n* = 901/1037) of patients with pre-index SLE versus 79.2% (*n* = 859/1084) of patients with newly confirmed SLE diagnosis having organ damage during follow-up. For patients with newly confirmed SLE diagnoses without pre-index organ damage, organ damage occurred for the first time in 24.0% (*n* = 260/1084) of patients during follow-up. All organ domains, except diabetes, were more frequently affected in the SLE cohort than the comparator cohort (Fig. 3). The high pre-index burden of organ damage was largely driven by ocular and neuropsychiatric damage (Fig. 3A). The largest absolute difference in percentage of patients with organ damage in the follow-up period between the SLE and comparator cohorts was observed in the cutaneous organ domain followed by the cardiovascular domain (Fig. 3B).

The risk of organ damage was slightly higher with corticosteroid treatment (OR [95% CI] 1.011 [1.004, 1.017]) than with any other medications, while a preventive tendency was seen with antimalarials (OR [95% CI] 0.989 [0.985, 0.993]) and methotrexate (OR [95% CI] 0.996 [0.992, 1.000]).

### Time to onset of organ damage in patients with SLE

**Fig. 4:**
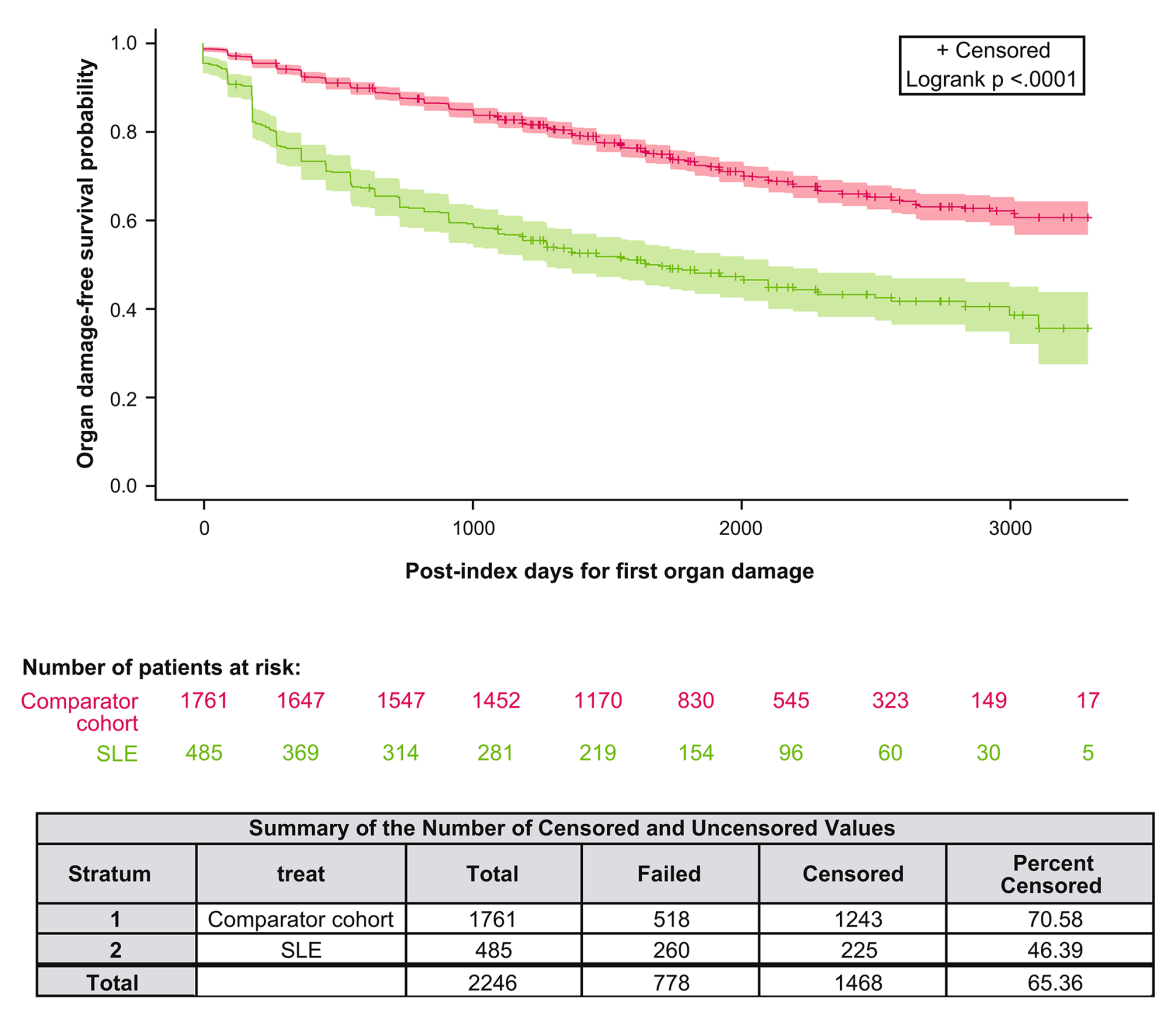
Kaplan–Meier curves of first occurrence of organ damage since the index date (in patients without organ damage before index) Kaplan–Meier curves represent the first occurrence of any organ damage in newly confirmed SLE diagnosis (*N* = 485) and comparator (*N* = 1761) patients without baseline organ involvement. The obvious significance of the differences is also confirmed by the log-rank test (*p* < 0.0001) SLE: systemic lupus erythematosus

In patients without pre-index organ damage, the first occurrence of any organ damage was significantly earlier in the newly confirmed SLE diagnosis cohort than in the patients in the comparator cohort (HR 2.578 [95% CI: 2.215, 3.001]; *p* < 0.0001) (Fig. 4).

The risk of organ damage (adjusted for sex, age, and baseline CCI comorbidities) in the newly confirmed SLE diagnosis cohort without pre-index organ damage was significantly higher for all organ domains versus the comparator cohort, except for gonadal involvement, diabetes, and malignancy (Table 2).

**Table 2:** Time to first organ damage for newly diagnosed SLE cohort without pre-index organ damage versus comparator cohort^a^

Organ domain	Parameter estimate	Standard error	*p* value	Hazard ratio (95% confidence interval)
Any organ damage	0.47348	0.03874	**< 0.0001**	2.578 (2.215–3.001)
Ocular	0.38422	0.06288	**< 0.0001**	2.156 (1.685–2.759)
Neuropsychiatric	0.42128	0.07576	**< 0.0001**	2.322 (1.726–3.125)
Renal	0.75135	0.13060	**< 0.0001**	4.494 (2.693–7.498)
Pulmonary	1.09683	0.20112	**< 0.0001**	8.968 (4.077–19.728)
Cardiovascular	0.56146	0.09545	**< 0.0001**	3.074 (2.114–4.469)
Peripheral vascular	0.65125	0.13699	**< 0.0001**	3.678 (2.150–6.293)
Gastrointestinal	0.27635	0.13122	**0.0352**	1.738 (1.039–2.907)
Musculoskeletal	0.69552	0.19325	**0.0003**	4.019 (1.884–8.572)
Cutaneous	1.71621	0.18933	**< 0.0001**	30.951 (14.736–65.012)
Gonadal	0.06367	0.14398	0.6584	1.136 (0.646–1.997)
Diabetes	0.10127	0.12263	0.4089	1.225 (0.757–1.980)
Malignancy	0.09435	0.11792	0.4236	1.208 (0.761–1.917)

^a^Results of an adjusted Cox regression

SLE: systemic lupus erythematosus

Patients with newly confirmed SLE diagnosis without organ damage at baseline were estimated to be nearly twice as likely to develop organ damage within 5 years as morbidity-matched comparator patients (52.0% [95% CI: 47, 56] vs. 27.0% [95% CI: 25, 29]). Probability estimations by organ domains in newly confirmed SLE diagnosis patients were also evaluated. Although the absolute probability of renal involvement within 5 years was only 7.0% in the newly confirmed SLE diagnosis cohort, it was higher than that of the comparator cohort (1.0%) (Table S5 in Additional file 1). Additionally, the 5-year probability estimate for ocular, neuropsychiatric, cutaneous, and cardiovascular organ domain involvement in the newly confirmed SLE diagnosis cohort was 18.0%, 14.0%, 13.0%, and 10.0%, respectively (Table S5).

### Time to worsening of organ damage in patients with pre-index SLE

In all SLE patients (with or without pre-index organ damage), the mean (standard error) time to worsening of any post-index organ damage was significantly earlier in the SLE cohort (4.0 years [0.078]) than in patients in the comparator cohort (5.6 years [0.044]) (HR 1.506 [95% CI: 1.387, 1.635]; *p* < 0.0001) (Fig. S4 in Additional file 1).

## Discussion

This analysis provides real-world data on the economic and clinical burden of organ damage in patients with SLE in Germany.

### Use of an organ damage claims algorithm

The organ damage claims algorithm utilized in this analysis allowed a comparison of the incremental organ damage burden expected to be attributable to SLE, as opposed to conditions included in the SDI that may occur regardless of an SLE diagnosis. In addition, the algorithm allowed cumulative organ damage to be objectively determined and applied to a broad population. We found organ damage increased over time and was consistently higher, and developed or progressed earlier, in patients with SLE than in comparator patients. Organ systems frequently affected were the ocular, cutaneous, neuropsychiatric, and cardiovascular domains. We also found that in patients without pre-index organ damage, the first occurrence of organ damage was significantly earlier in patients with a newly confirmed SLE diagnosis than in the comparator cohort; this could be explained by the more careful evaluation of patients with a newly confirmed SLE diagnosis. The risk of organ damage was greater for patients with a newly confirmed SLE diagnosis compared with comparators in all organ domains apart from gonadal involvement, diabetes, and malignancy. The increase in organ damage over time and higher rate of occurrence in the SLE cohort versus a comparator cohort parallels the findings of a similar claims study in Taiwan, which reported that > 80% of patients with SLE developed organ damage within 6 months of diagnosis. It should be noted that this high estimate may be due to the definition of organ damage as ≥ 3 separate records of an ICD-9 code, which could be achieved by frequent patient visits in the first 6 months [17]. In contrast, our analysis followed the SDI requirement that organ damage manifestations be present for ≥ 6 months in order to be considered irreversible [26, 27]. A recent study in the USA, which applied the same organ damage claims algorithm utilized here but adapted to the US ICD system, identified similar most-affected organ systems to those our study identified, with neuropsychiatric, ocular, and cardiovascular the most common sites of organ damage [16]. Findings from a qualitative interview study also suggest SLE with organ damage has a more severe and debilitating impact on all aspects of patients’ lives than SLE prior to organ damage development, highlighting the important burden of organ damage to patients [28].

### Economic burden and HCRU associated with organ damage

Our findings show that annual costs were significantly higher for patients with SLE versus comparators and remained consistently higher over time. In total, 83.0% of patients with SLE presented with new organ damage during the study period, which was associated with significant economic burden. The additional cost and HCRU burden was also considerably greater in patients with SLE with organ damage than in those without organ damage.

A number of previous studies have evaluated the costs associated with organ damage in SLE [15–21]; however, findings from European populations are limited. Our findings on the economic burden of SLE with organ damage are similar to the results of other cost analyses; we found the economic burden of patients with SLE and organ damage exceeded that of patients with SLE without organ damage, and of comparator patients. This finding is consistent with a previous study on a similar European population that found organ damage to be a significant predictor of direct cost, but also that organ damage, and not disease activity, predicted increased indirect costs [19]. In agreement with previous findings [29], the total costs of patients with SLE in this study were mainly driven by inpatient admissions and pharmacy costs. During the follow-up period, among patients with SLE with organ damage, the number of inpatient admissions and length of stay were higher than among those without organ damage.

In this analysis, over half of the patients with newly confirmed SLE diagnoses had pre-index organ damage. Organ damage can exist for several years prior to a formal SLE diagnosis [30]; however, in most clinical studies and by definition in the SDI, organ damage is captured only after SLE diagnosis (an exception being the SLICC cohort study, which captured organ damage at inception) [7, 11]. Nevertheless, the costs of SLE-associated organ damage are relevant to the disease burden and to the payer perspective whether the organ damage condition began before or after the definitive SLE diagnosis. In addition, SDI is not consistently measured in routine clinical practice [31]. Analyses using secondary data sources mean that, while SDI is not recorded, information describing organ damage is available throughout the patient journey, including prior to SLE diagnosis. Therefore, in contrast to the clinical use of the SDI, organ damage that preceded the index date (first SLE diagnosis code) was included in this analysis of economic burden. In SLE specifically, delays before definitive diagnosis are common due to the heterogeneous presentation of the disease [32]. Considering that existing organ damage is a key risk factor for organ damage accrual [11, 27, 33–36], these potential delays are of concern, as pre-existing organ damage before diagnosis may not be captured or recognized. In addition, only a specific timeframe is available in our database, which is different to a clinical setting, where electronic health records are available and information can be requested from the patients directly. Therefore, we considered a record of organ damage before SLE diagnosis to be of relevance.

The potentially modifiable risk factors for organ damage are uncontrolled disease activity, including recurrence of severe flares, exposure to glucocorticoids and/or immunosuppressive therapies [3, 4, 11, 34, 37–40], and the number of attending physicians [41]. Drugs that treat the underlying disease mechanism and modify the course of SLE may slow or prevent the progression of organ damage by controlling disease activity. Currently recommended treatments for SLE include antimalarials, immunosuppressive agents, glucocorticoids, and biologics [8]. Similar to other real-world studies [7, 37, 38, 42, 43], in our SLE cohort, despite EULAR guideline recommendations [8], not all patients were prescribed antimalarials or appropriate dosing of glucocorticoids. In an exploratory analysis of the current study, higher cumulative steroid dose increased the odds of SDI worsening, while a preventive tendency was seen with longer duration of antimalarials and methotrexate treatment. Given the economic burden of organ damage highlighted in the current study, additional emphasis on the importance of slowing or preventing organ damage is warranted. For instance, the higher disease burden of patients with organ damage versus no organ damage is reflected in pharmacy costs in this study, which were greater in patients with SLE with organ damage compared with those without organ damage.

### Limitations

Limitations of the current study include the absence of clinical and disease activity information within the claims data, due to the claims database being designed for reimbursement. Further, the true date of diagnosis can be difficult to capture, as the timing of outpatient diagnoses is accurate only to a quarter of a year, initial physicians’ diagnosis of SLE may be miscoded, visits to specialists can be delayed, and initial diagnosis could have been made before the data of the database is available. Due to these limitations of capturing the true diagnosis date, the exact date of organ damage and disease duration cannot be determined, which is an inherent limitation of claims data. However, our organ damage claims algorithm required organ damage symptoms to persist for least 6 months to robustly capture irreversible damage. Therefore, we believe that despite the limitations around capturing the exact diagnosis and organ damage dates, our study provides valuable information on prevalence and progression of organ damage over time. The assessment of economic burden relied on information provided by the healthcare payers (direct costs) and sickness pay, but did not reflect other indirect costs such as productivity loss (although data were available for work disability); it has been demonstrated previously that indirect costs of SLE are more than twice that of direct costs [19]. In addition, while the duration of follow-up for newly confirmed SLE cases was maximized as far as possible in the data source, it may still be too short to fully capture organ damage that presents over the longer term. Similarly, the 2-year pre-index period may not have been long enough to accurately classify some patients with a newly confirmed diagnosis of SLE, and due to the inclusion of pre-index organ damage, some pre-index conditions may have been misattributed to SLE-related organ damage rather than comorbid conditions. We acknowledge that race and ethnicity could be important matching characteristics; however, data on race and ethnicity, and sociodemographic information, were not available as they were not recorded in the database used. Therefore, given the demographics in Germany, it may be difficult to generalize these findings to countries with more racially diverse populations. It has also been shown in a prospective cohort study that African-American patients with SLE accrue more organ damage, more quickly, compared with White patients with SLE, with differences in individual organ domains due to both ethnicity and socioeconomic factors [44]. Patients with organ damage were older than patients without organ damage, which could have been a confounder in the cost comparison; however, sensitivity analysis matching the two subgroups by age and sex demonstrated Year 1 costs in patients with organ damage were 2.07 times that of patients without organ damage, similar to the factor of 2.18 in the unmatched subgroups. Finally, propensity matching by CCI comorbidities meant that the true burden of SLE-associated organ damage may have been underestimated, since our comparator cohort had a high comorbidity burden as opposed to being a healthy, or even general population, comparator group. For example, 52.7% of the comparator cohort had rheumatologic conditions other than SLE pre-index, some of which (e.g. rheumatoid arthritis) are also associated with organ damage, such as cardiovascular disease [45]. Nevertheless, this approach was taken in order to investigate the impact of SLE alone, compared with the comparator population, accepting that the comparator group had more comorbidities than the general population. Furthermore, the overlap between conditions in the CCI and organ damage, aside from the circular argument that more organ damage will by definition be reflected as higher CCI score, also results in a further potential overestimation of organ damage in the comparator and SLE cohorts, as they were matched for CCI and thus may have potentially matched for organ damage conditions as well. Finally, it is also noteworthy to mention that the reporting of medications was based on dispensed medications, with no certainty that the patient took their medication as prescribed.

## Conclusions

In summary, based on this recently developed organ damage claims algorithm, utilizing a real-world claims database, it was possible to determine the burden of organ damage in a large representative sample of patients with SLE in the real-world setting in Germany. The clinical burden of organ damage was high in patients with SLE, and this was associated with a significant economic burden. A substantial proportion of patients with SLE in Germany have existing organ damage at diagnosis. This algorithm has the potential to be applied in studies where SDI was not measured, and could be used to make outcomes more comparable in future claims data studies. Overall, these results highlight the importance of early SLE diagnosis and intervention with disease-modifying therapies to minimize disease activity and slow or prevent organ damage progression to improve outcomes and reduce the economic burden of SLE.

### Electronic supplementary material

Below is the link to the electronic supplementary material.

Supplementary Material 1

## Data Availability

The datasets generated and/or analyzed during the current study are not publicly available due to data protection reasons but are available from the corresponding author on reasonable request (only derived/ aggregated datasets for temporary use).

## Abbreviations

ACR: American College of Rheumatology
BKK: Betriebskrankenkassen
CCI: Charlson comorbidity index
CI: Confidence interval
EULAR: European Alliance of Associations for Rheumatology
GKV: Gesetzliche Krankenversicherung
HCRU: Healthcare resource utilization
HR: Hazard ratio
ICD-10-GM: International Classification of Diseases,10th Revision, German Modification
OR: Odds ratio
PY: Patient-year
SD: Standard deviation
SDI: SLICC/ACR Damage Index
SLE: Systemic lupus erythematosus
SLICC: Systemic Lupus International Collaborating Clinics
