# Supplementary material for: Clinical and economic burden of organ damage among patients with systemic lupus erythematosus in a real-world setting in Germany
Source: BMC Rheumatol. 2024 May 17;8:18. doi: 10.1186/s41927-024-00387-6 (PMC11100138; doi:10.1186/s41927-024-00387-6)
Supplement: Supplementary file 1 — Supplementary Material 1 [file 41927_2024_387_MOESM1_ESM.docx]

## Additional file 1. Supplementary materials

**Fig. S1. Identification and validation of study cohorts**

**
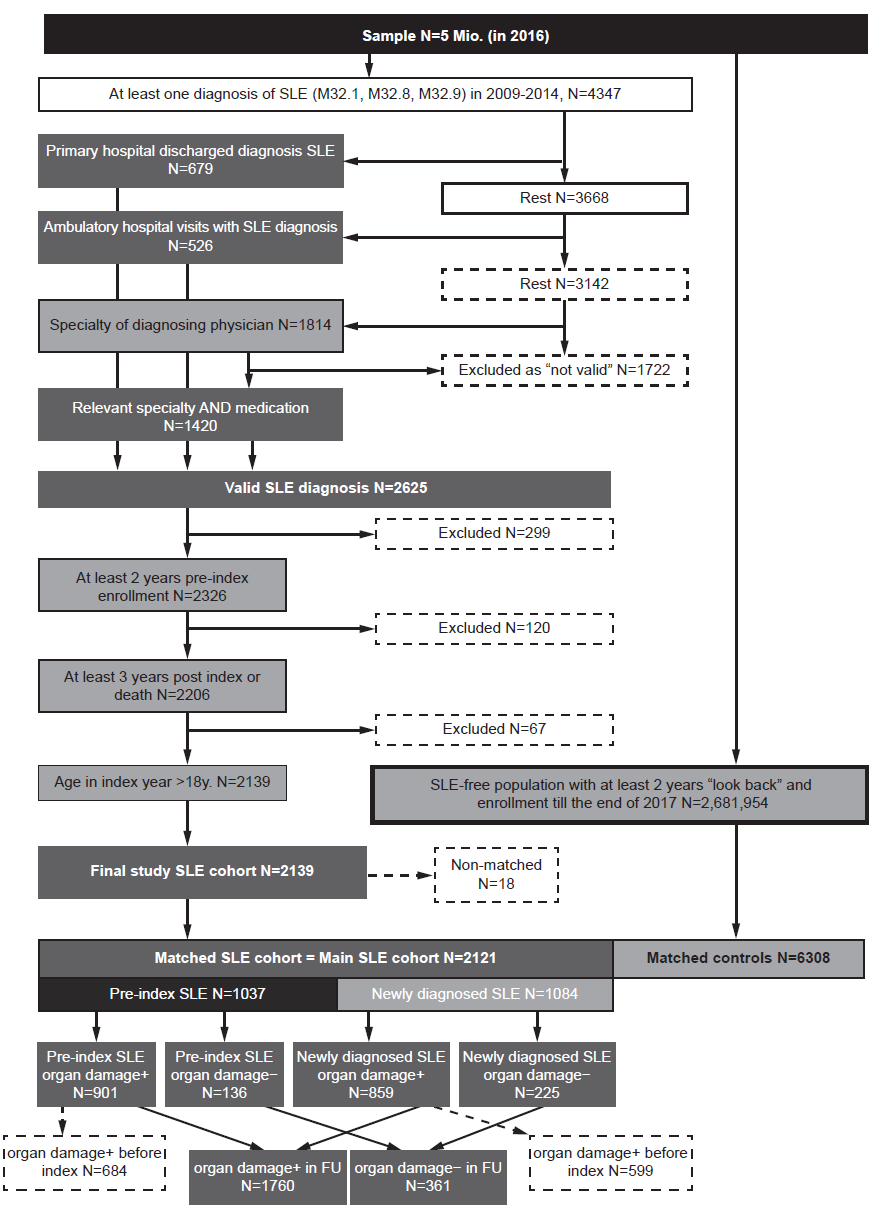
**

FU, follow-up; SLE, systemic lupus erythematosus.

### HCRU results

The number of outpatient visits per patient-year was also higher for the SLE with organ damage subgroup than for the SLE without organ damage subgroup (**Table S2**). For the outpatient visits, the factors of with/without organ damage generally grew consistently with time: from 1.50 (Year 1) to 1.89 (Year 6) for the number of outpatient visits per patient-year and from 1.73 to 2.42 for the associated costs (**Figure S2** and **Table S2**).

The differences in ambulatory visits and the associated costs between SLE with organ damage and SLE without organ damage subgroups were not as pronounced as for other HCRUs (**Table S2** and **Figure S2**).

The number of pharmacy claims was higher in the SLE with organ damage subgroup compared with the SLE without organ damage subgroup (**Table S2**). The pharmacy costs per patient-year in the SLE with organ damage subgroup exceeded the corresponding costs in the SLE without organ damage subgroup by a factor ranging from 1.74 (Year 4) to 2.87 (Year 6; **Figure S2**). Regarding long-term work disability, the costs were consistently higher in the SLE with organ damage subgroup than in the SLE without organ damage subgroup (**Table S4**). While the days of work disability were slightly higher in the SLE without organ damage versus SLE with organ damage subgroup in Years 4 and 6, the days of long-term (>42 days) work disability were higher in the SLE with organ damage subgroup versus SLE without organ damage subgroup throughout all follow-up years (**Table S4**). This may be due to the SLE without organ damage subgroup being younger (SLE subgroups were not matched by age), with potentially more patients of employable age in the SLE without organ damage subgroup having days of work disability recorded than in the older SLE with organ damage subgroup.

The differences in costs for additional aids and services, including transportation costs, rehabilitation, occupational therapy, physiotherapy and basic and household nursing, were substantially pronounced between the SLE with organ damage and SLE without organ damage subgroups. The with/without organ damage ratio varied between 4.32 and 7.75. The highest cost per patient-year of €1167.74 (Year 4) was observed in the SLE with organ damage subgroup, while in the SLE without organ damage subgroup, the highest cost was €211.56 (Year 5).

In total, 22.8% (n=483) of patients with SLE underwent an organ damage–relevant medical procedure during follow-up, compared with 17.3% (n=1092) of patients in the comparator population, with the most common procedure being cataract surgery (SLE population, 10.7%; comparator population: 9.9%). Significantly more patients with SLE underwent membranectomy/vitrectomy, had admissions for dialysis, and cardiovascular, angioplasty, pericardiectomy, and gastrointestinal procedures than the comparator population.

## Fig. S2. Mean annual costs of HCRU per patient-year for patients with and without organ damage in the follow-up period (SLE cohort, N=2121)


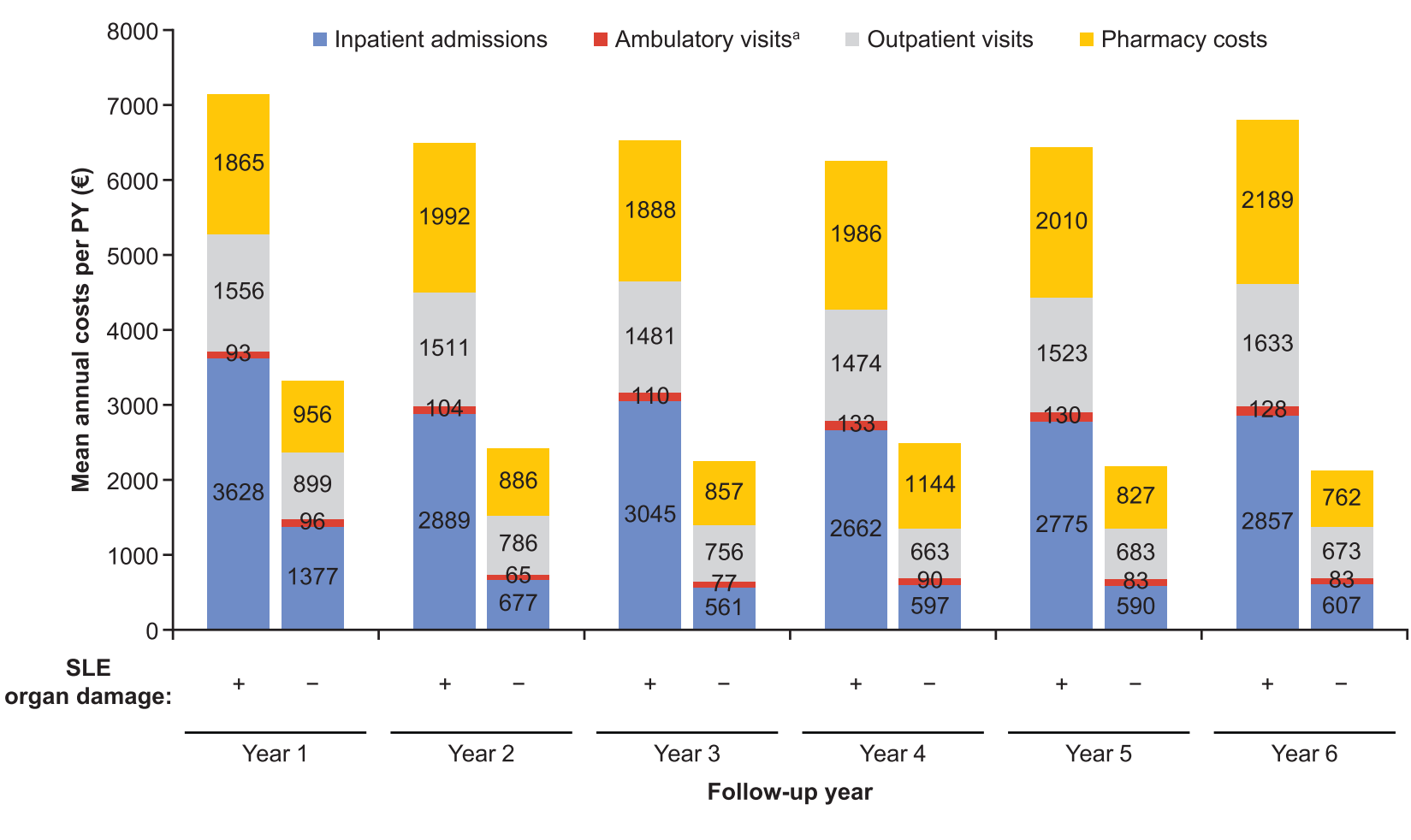


^a^Includes all walk-in hospital health services without an in-bed stay.
HCRU, healthcare resource utilization; SLE, systemic lupus erythematosus.

## Fig. S3. Cumulative duration^a^ of SLE medication use per patient-year in days by drug class (SLE cohort, N=2121)


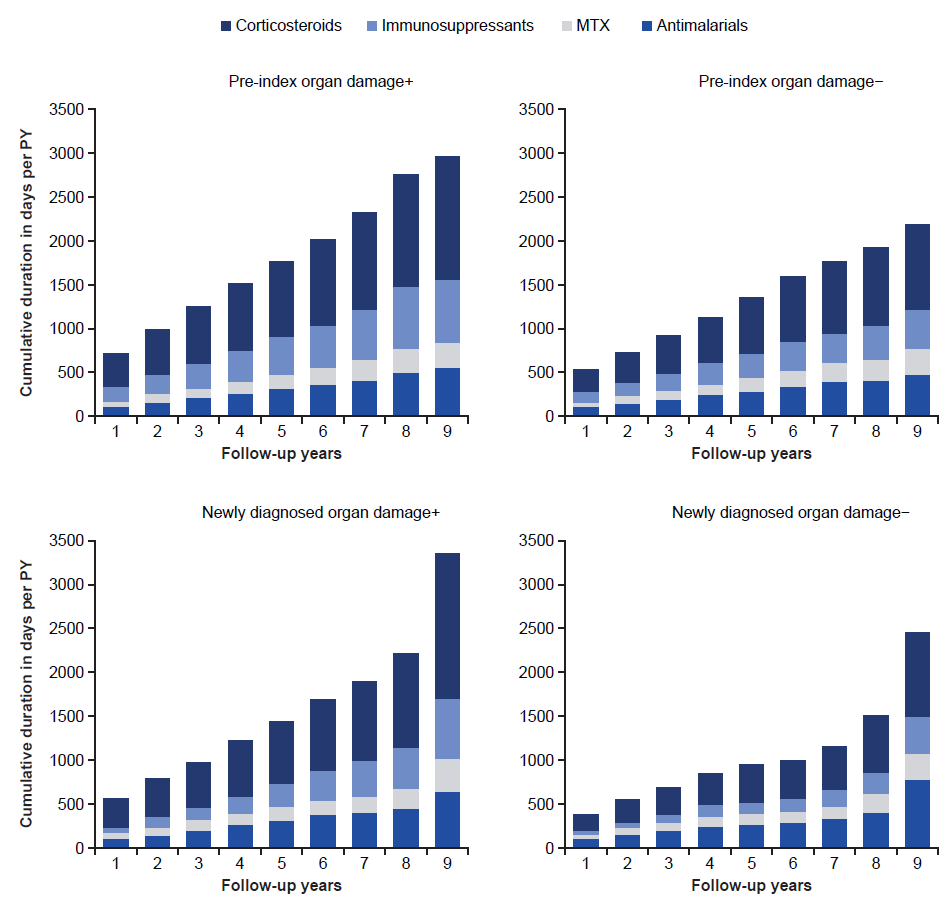


^a^Cumulative duration for different groups of medications used in SLE was estimated based on Defined Daily Dose (DDD) in the entire study period. The full dose per pharmacy was calculated using information about the dosage and package contained in ‘Pharmazentralnummer’. The ratio of the full dose to DDD corresponded to the duration of every prescription. For the calculation of the cumulative duration per year, the duration of prescription carrying over into the next year was considered proportional to the days of intake in the reference year. The cumulative duration of systemic corticosteroids estimated did not reflect a real-time medication use due to the wide variability of the dosages used in SLE depending on disease activity and severity (actual prescribed corticosteroid doses vary from 1 to 100 mg per tablet or up to 1000 mg intravenously). Therefore, the cumulative dosage estimated for corticosteroids was based on the prednisone-equivalent dosage (DDD=10 mg), which was expected to be more relevant to organ damage.

MTX, methotrexate; SLE, systemic lupus erythematosus.

**Fig. S4. Kaplan–Meier curves for the first worsening of organ damage since the index date in the SLE cohort and comparator cohort**


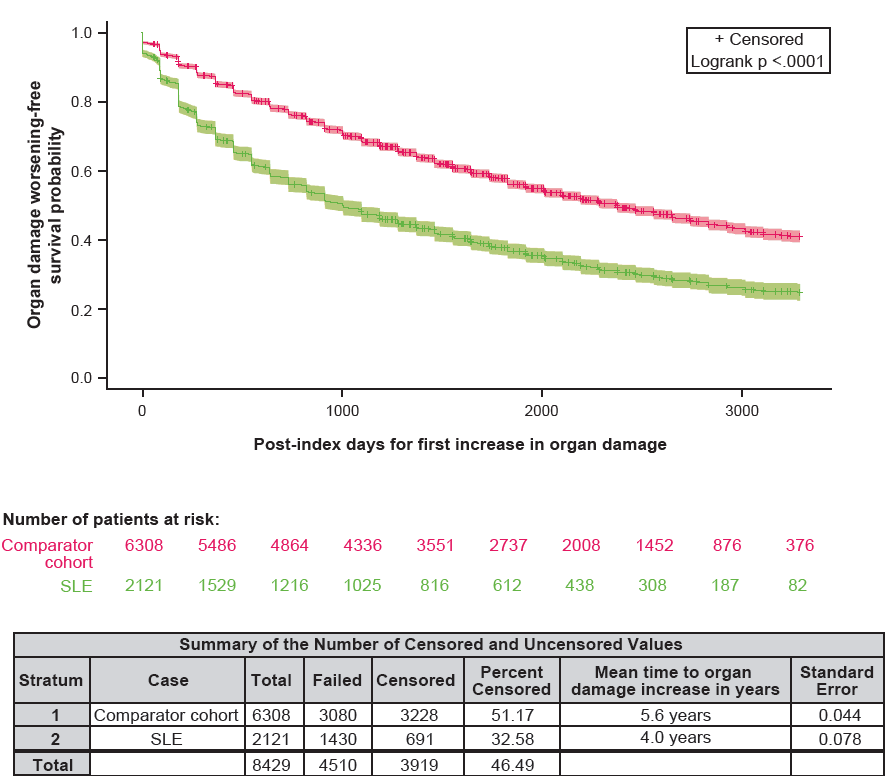


Kaplan–Meier curves represent the first occurrence of any worsening of organ damage since the index date in SLE (N=2121) and comparator (N=6308) patients. The obvious significance of the differences is also confirmed by the log-rank test (p<0.0001).

SLE, systemic lupus erythematosus.

**Tables**

## Table S1. Baseline characteristics for patients with and without organ damage (SLE cohort, N=2121)

|  | **Organ damage+**  **(N=1760)** | | **Organ damage−**  **(N=361)** | |
| --- | --- | --- | --- | --- |
| Female, n (%) | 1461 (83.01) | | 309 (85.60) | |
| Age, mean (SD) | 53.41 (15.92) | | 38.43 (10.83) | |
| Follow-up length, mean (SD), years | 6.40 (2.08) | | 6.23 (1.86) | |
| CCI score, mean (SD) | 2.21 (1.99) | | 0.85 (0.94) | |
| **Organ domain involvement in the SLE cohort^a^, n (%)** | | | | |
| Any organ damage | | 1283 (60.49) | | - |
| Ocular | | 570 (26.87) | | - |
| Neuropsychiatric | | 400 (18.86) | | - |
| Renal | | 139 (6.55) | | - |
| Pulmonary | | 99 (4.67) | | - |
| Cardiovascular | | 308 (14.52) | | - |
| Peripheral vascular | | 173 (8.16) | | - |
| Gastrointestinal | | 61 (2.88) | | - |
| Musculoskeletal | | 74 (3.49) | | - |
| Cutaneous | | 178 (8.39) | | - |
| Gonadal | | 52 (2.45) | | **-** |
| Diabetes | | 241 (11.36) | | **-** |
| Malignancy | | 194 (9.15) | | **-** |
| **Baseline SLE medications^a,b^, n (%)** |  | |  | |
| Antimalarials | 841 (47.8) | | 181 (50.1) | |
| Corticosteroids | 1245 (70.7) | | 208 (57.6) | |
| Immunosuppressants | 518 (29.4) | | 70 (19.4) | |
| Methotrexate | 249 (14.2) | | 47 (13.0) | |
| Cyclophosphamide | 30 (1.7) | | 2 (0.6) | |
| Sulfasalazine | 18 (1.0) | | 3 (0.8) | |
| Biologics | 17 (1.0) | | 2 (0.6) | |
| Belimumab | 4 (0.2) | | 2 (0.6) | |

^a^Percentage of the SLE cohort (n=2121)

^b^For the patients with pre-index SLE, number of patients receiving SLE treatment was assessed since the first SLE diagnosis documented in the pre-index period and during the first 12 months after index date; for patients with newly diagnosed SLE, numbers of patients receving SLE treatment was assessed during the first 12 months after the index date.

CCI, Charlson comorbidity index; SD, standard deviation; SLE, systemic lupus erythematosus.

## Table S2. HCRU among patients with and without organ damage

|  | **Year 1** | **Year 2** | **Year 3** | **Year 4** | **Year 5** | **Year 6** |
| --- | --- | --- | --- | --- | --- | --- |
| **Number of inpatient admissions per patient-year** | | | | | | |
| **SLE organ damage+** | 0.97 | 0.70 | 0.65 | 0.61 | 0.64 | 0.64 |
| **SLE organ damage−** | 0.46 | 0.24 | 0.23 | 0.21 | 0.19 | 0.20 |
| **Pre-index SLE organ damage+** | 0.78 | 0.65 | 0.65 | 0.65 | 0.66 | 0.64 |
| **Pre-index SLE organ damage−** | 0.34 | 0.25 | 0.29 | 0.26 | 0.17 | 0.20 |
| **Newly diagnosed SLE organ damage+** | 1.18 | 0.76 | 0.64 | 0.57 | 0.61 | 0.63 |
| **Newly diagnosed SLE organ damage−** | 0.53 | 0.23 | 0.19 | 0.17 | 0.21 | 0.20 |
| **Length of inpatient admission per patient-year** | | | | | | |
| **SLE organ damage+** | 9.65 | 6.81 | 6.93 | 6.34 | 6.60 | 6.11 |
| **SLE organ damage−** | 4.36 | 1.82 | 1.61 | 1.83 | 1.71 | 1.23 |
| **Pre-index SLE organ damage+** | 7.28 | 6.31 | 6.86 | 6.21 | 7.00 | 6.48 |
| **Pre-index SLE organ damage−** | 2.61 | 2.43 | 2.11 | 2.82 | 2.03 | 1.22 |
| **Newly diagnosed SLE organ damage+** | 12.14 | 7.34 | 7.01 | 6.50 | 6.05 | 5.44 |
| **Newly diagnosed SLE organ damage−** | 5.42 | 1.44 | 1.30 | 1.18 | 1.47 | 1.25 |
| **Number of ambulatory visits^a^ per patient-year** | | | | | | |
| **SLE organ damage+** | 0.57 | 0.58 | 0.69 | 0.77 | 0.81 | 0.83 |
| **SLE organ damage−** | 0.74 | 0.55 | 0.61 | 0.65 | 0.64 | 0.57 |
| **Pre-index SLE organ damage+** | 0.29 | 0.42 | 0.65 | 0.81 | 0.83 | 0.89 |
| **Pre-index SLE organ damage−** | 0.26 | 0.26 | 0.34 | 0.46 | 0.59 | 0.50 |
| **Newly diagnosed SLE organ damage+** | 0.87 | 0.76 | 0.73 | 0.73 | 0.79 | 0.73 |
| **Newly diagnosed SLE organ damage−** | 1.03 | 0.73 | 0.78 | 0.77 | 0.68 | 0.65 |
| **Number of outpatient visits per patient-year** | | | | | | |
| **SLE organ damage+** | 44.24 | 40.27 | 38.35 | 37.91 | 38.38 | 38.57 |
| **SLE organ damage−** | 29.52 | 24.04 | 22.64 | 20.75 | 21.02 | 20.36 |
| **Pre-index SLE organ damage+** | 42.70 | 41.27 | 39.17 | 40.00 | 40.18 | 40.17 |
| **Pre-index SLE organ damage−** | 26.91 | 22.85 | 22.51 | 20.50 | 21.74 | 20.03 |
| **Newly diagnosed SLE organ damage+** | 45.85 | 39.22 | 37.47 | 35.50 | 35.86 | 35.65 |
| **Newly diagnosed SLE organ damage−** | 31.10 | 24.77 | 22.71 | 20.91 | 20.47 | 20.69 |
| **Number of pharmacy claims per patient-year** | | | | | | |
| **SLE organ damage+** | 19.25 | 19.01 | 18.75 | 18.85 | 18.84 | 19.19 |
| **SLE organ damage−** | 9.63 | 8.17 | 7.77 | 7.49 | 7.16 | 7.23 |
| **Pre-index SLE organ damage+** | 19.18 | 19.30 | 19.23 | 20.05 | 19.99 | 20.38 |
| **Pre-index SLE organ damage−** | 9.31 | 8.40 | 7.89 | 7.67 | 7.78 | 7.28 |
| **Newly diagnosed SLE organ damage+** | 19.33 | 18.70 | 18.24 | 17.47 | 17.25 | 17.03 |
| **Newly diagnosed SLE organ damage−** | 9.82 | 8.04 | 7.70 | 7.37 | 6.68 | 7.19 |

^a^Includes all walk-in hospital health services without an in-bed stay.
HCRU, healthcare resource utilization; SLE, systemic lupus erythematosus.

## Table S3. Reasons for inpatient admissions in the overall SLE population (N=1691)

| **3-character ICD-10-GM** | **Number of cases** | **Number of patients (% of all with utilizations)** |
| --- | --- | --- |
| M32 – SLE | 1525 | 565 (33.4) |
| M35 – other systemic involvement of connective tissue | 246 | 116 (6.9) |
| L93 – lupus erythematosus | 203 | 97 (5.7) |
| J18 – pneumonia, organism unspecified | 113 | 92 (5.4) |
| I50 – heart failure | 136 | 88 (5.2) |
| I20 – angina pectoris | 90 | 71 (4.2) |
| I21 – acute myocardial infarction | 78 | 60 (3.5) |
| M54 - dorsalgia | 79 | 60 (3.5) |
| K80 - cholelithiasis | 80 | 59 (3.5) |
| I10 – essential (primary) hypertension | 73 | 58 (3.4) |
| I63 – cerebral infarction | 76 | 57 (3.4) |
| K29 – gastritis and duodenitis | 57 | 57 (3.4) |
| A41 – other sepsis | 70 | 53 (3.1) |
| M17 – gonarthrosis (arthrosis of knee) | 72 | 53 (3.1) |
| I70 - atherosclerosis | 105 | 52 (3.1) |
| N18 – chronic kidney disease | 97 | 52 (3.1) |
| J44 – other chronic obstructive pulmonary disease | 74 | 49 (2.9) |
| N39 – other disorders of urinary system | 56 | 49 (2.9) |
| M79 – other soft tissue disorders, not elsewhere classified | 61 | 48 (2.8) |
| I25 – chronic ischaemic heart disease | 66 | 47 (2.8) |

ICD-10-GM, International Classification of Diseases, 10th Revision, German Modification; SLE, systemic lupus erythematosus.

## Table S4. Costs (€) and days of work disability per patient-year by follow-up year

|  | **Year 1** | **Year 2** | **Year 3** | **Year 4** | **Year 5** | **Year 6** |
| --- | --- | --- | --- | --- | --- | --- |
| **Costs of long-term disability per patient-year** | | | | | | |
| **SLE organ damage+** | 456.40 | 234.40 | 270.17 | 257.76 | 347.02 | 235.72 |
| **SLE organ damage−** | 319.18 | 49.61 | 108.10 | 226.31 | 192.12 | 211.64 |
| **Pre-index SLE organ damage+** | 217.79 | 169.96 | 176.73 | 203.14 | 449.50 | 221.57 |
| **Pre-index SLE organ damage−** | 268.09 | 67.13 | 90.00 | 384.18 | 107.75 | 332.76 |
| **Newly diagnosed SLE organ damage+** | 656.95 | 278.13 | 347.10 | 301.75 | 181.78 | 248.55 |
| **Newly diagnosed SLE organ damage−** | 324.43 | 35.09 | 111.74 | 112.84 | 249.19 | 78.09 |
| **Sum days of work disability per patient-year** | | | | | | |
| **SLE organ damage+** | 21.22 | 14.71 | 14.91 | 13.37 | 14.58 | 11.98 |
| **SLE organ damage−** | 18.48 | 10.98 | 11.73 | 15.39 | 14.21 | 12.54 |
| **Pre-index SLE organ damage+** | 14.13 | 12.67 | 12.66 | 13.55 | 16.67 | 11.13 |
| **Pre-index SLE organ damage−** | 20.09 | 11.81 | 9.69 | 18.71 | 11.82 | 13.87 |
| **Newly diagnosed SLE organ damage+** | 28.67 | 16.86 | 17.31 | 13.17 | 11.66 | 13.52 |
| **Newly diagnosed SLE organ damage−** | 17.50 | 10.47 | 12.97 | 13.23 | 16.07 | 11.17 |
| **Sum days of long-term disability** | | | | | | |
| **SLE organ damage+** | 14.26 | 8.48 | 8.65 | 7.72 | 8.44 | 6.49 |
| **SLE organ damage−** | 10.98 | 3.45 | 3.90 | 7.42 | 6.38 | 4.50 |
| **Pre-index SLE organ damage+** | 8.06 | 6.79 | 6.62 | 7.69 | 10.61 | 6.27 |
| **Pre-index SLE organ damage−** | 14.63 | 4.27 | 2.97 | 12.25 | 5.57 | 6.99 |
| **Newly diagnosed SLE organ damage+** | 20.79 | 10.27 | 10.82 | 7.76 | 5.41 | 6.88 |
| **Newly diagnosed SLE organ damage−** | 8.77 | 2.95 | 4.46 | 4.30 | 7.00 | 1.93 |

SLE, systemic lupus erythematosus.

**Table S5**. **Year 5 estimates of probability of organ damage by organ domains in newly diagnosed SLE and comparator cohorts, without patients with organ damage before index**

| **Organ domain** | **Cohort** | **Organ damage free** | **Organ damage rate** | **Organ damage rate**  **[95% CI]** | **Survival standard error** | **Number failed** | **Number left** |
| --- | --- | --- | --- | --- | --- | --- | --- |
| **Ocular** | Comparator | 0.8996 | 0.1004 | [0.09, 0.12] | 0.00755 | 163 | 955 |
|  | SLE | 0.8172 | 0.1828 | [0.15, 0.22] | 0.0181 | 85 | 235 |
| **Neuropsychiatric** | Comparator | 0.9365 | 0.0635 | [0.05, 0.08] | 0.00612 | 103 | 994 |
|  | SLE | 0.8579 | 0.1421 | [0.11, 0.17] | 0.0167 | 64 | 249 |
| **Renal** | Comparator | 0.9879 | 0.0121 | [0.01, 0.02] | 0.00289 | 18 | 1048 |
|  | SLE | 0.9316 | 0.0684 | [0.04, 0.09] | 0.0120 | 31 | 272 |
| **Pulmonary** | Comparator | 0.9953 | 0.00471 | [0.00, 0.01] | 0.00181 | 7 | 1055 |
|  | SLE | 0.9644 | 0.0356 | [0.02, 0.05] | 0.00848 | 17 | 280 |
| **Cardiovascular** | Comparator | 0.9644 | 0.0356 | [0.03, 0.04] | 0.00478 | 55 | 1,024 |
|  | SLE | 0.9046 | 0.0954 | [0.07, 0.12] | 0.0138 | 44 | 270 |
| **Peripheral vascular** | Comparator | 0.9847 | 0.0153 | [0.01, 0.02] | 0.00305 | 25 | 1046 |
|  | SLE | 0.9508 | 0.0492 | [0.03, 0.07] | 0.0100 | 23 | 276 |
| **Gastrointestinal** | Comparator | 0.9755 | 0.0245 | [0.02, 0.03] | 0.00393 | 39 | 1037 |
|  | SLE | 0.9585 | 0.0415 | [0.02, 0.06] | 0.00938 | 19 | 284 |
| **Musculoskeletal** | Comparator | 0.9925 | 0.00753 | [0.00, 0.01] | 0.00219 | 12 | 1051 |
|  | SLE | 0.9755 | 0.0245 | [0.01, 0.04] | 0.00735 | 11 | 285 |
| **Cutaneous** | Comparator | 0.9952 | 0.00480 | [0.00, 0.01] | 0.00170 | 8 | 1056 |
|  | SLE | 0.8743 | 0.1257 | [0.10, 0.16] | 0.0152 | 60 | 254 |
| **Gonadal** | Comparator | 0.9751 | 0.0249 | [0.02, 0.03] | 0.00394 | 40 | 1033 |
|  | SLE | 0.9655 | 0.0345 | [0.02, 0.05] | 0.00852 | 16 | 280 |
| **Diabetes** | Comparator | 0.9638 | 0.0362 | [0.03, 0.05] | 0.00466 | 59 | 1025 |
|  | SLE | 0.9553 | 0.0447 | [0.03, 0.06] | 0.00990 | 20 | 275 |
| **Malignancy** | Comparator | 0.9630 | 0.0370 | [0.03, 0.05] | 0.00473 | 60 | 1024 |
|  | SLE | 0.9440 | 0.0560 | [0.03, 0.08] | 0.0113 | 24 | 274 |

CI, confidence interval; SLE, systemic lupus erythematosus.
